# Oral health related quality of life of patients with class III skeletal malocclusion before and after orthognathic surgery

**DOI:** 10.1186/s12903-019-0989-9

**Published:** 2019-12-21

**Authors:** Farzad Rezaei, Hiwa Masalehi, Amin Golshah, Mohammad Moslem Imani

**Affiliations:** 1 Department of Oral and Maxillofacial Surgery, Kermanshah University of Medical Sciences, Kermanshah, Iran; 2 Students Research Committee, Kermanshah University of Medical Sciences, Kermanshah, Iran; 3 Department of Orthodontics, Kermanshah University of Medical Sciences, Kermanshah, Iran

**Keywords:** Class III malocclusion, Orthognathic surgery, Quality of life

## Abstract

**Background:**

Orthognathic surgery includes improvement of morphology and function of occlusion as well as psychological perception and oral health-related quality of life (OHRQoL) of patients. The aim of this study was to determine the OHRQoL of patients with class III skeletal malocclusion before and after orthognathic surgery.

**Materials and methods:**

A total of 112 skeletal class III patients including 39 (34.8%) males and 73 (65.2%) females participated in this descriptive quasi-experimental study in three groups: “prior to orthodontic treatment” (*n* = 25); “under orthodontic treatment and prior to surgery” (*n* = 65), and “after surgery” (n = 25). All patients filled out a demographic information questionnaire, the oral health impact profile-14 (OHIP-14), and the orthognathic quality of life questionnaire (OQLQ) under the supervision of the examiner. Data were analyzed using ANOVA, independent samples t-test, Mann Whitney test, and Kruskal-Wallis test.

**Results:**

OHRQoL summary score changed from 14.5 prior to orthodontic treatment to 23.4 prior to surgery and during orthodontic treatment to 5.4 after surgery. These OHRQoL changes were statistically significant (*P* < 0.001).

**Conclusions:**

Orthognathic surgery matters to patients with class III skeletal malocclusion and significantly improves their OHRQoL.

## Highlights

1. The OHRQoL of patients with class III skeletal malocclusion significantly deteriorated after orthodontic treatment and before the surgery compared with baseline preoperative state.
2. Orthognathic surgery significantly improves the OHRQoL in skeletal class III patients.

## Background

Facial esthetics has always been a major demand for patients. Despite different opinions in this respect, researchers have always been in search of a specific definition for a normal and pleasant appearance [1]. Some proportions have been proposed to define a beautiful face. However, these proportions may change over time and vary in different races and ethnic groups [1].

Unesthetic appearance can decrease the self-esteem of individuals [2]. Dentoskeletal malformations cause not only esthetic problems for patients but also can lead to psychosocial consequences. They are usually associated with impaired masticatory function and speech. Clinically, class III malocclusion is defined as retrognathism of the maxilla or prognathism of the mandible or a combination of both. The prevalence of class III malocclusion is higher in Asian populations compared with Caucasians (23% versus 5%) [3]. Class III malocclusion is often associated with complex dentoalveolar problems including the edge to edge position of teeth or posterior cross-bite. Class III patients mostly have esthetic problems, a concave profile and vertical functional pattern, which limits the function to vertical movements [3].

Despite the attempts of orthodontists to non-surgically treat patients with mandibular prognathism, a large number of these patients will eventually require orthognathic surgery. Orthodontic treatment of class III skeletal discrepancy includes three different therapeutic approaches namely (I) growth modification treatments performed in the pre-pubertal stage, (II) camouflage treatments performed after the growth spurt, and (III) removal of dental compensation to prepare the patient for surgery [1]. If the malocclusion cannot be well corrected non-surgically, orthognathic surgery will be the only treatment option left for the patient. Orthognathic surgery is a reliable approach for treatment of dentomaxillofacial deformities. It is performed aiming to improve esthetics, function, facial appearance, mastication and speech [4]. Aside from these advantages, orthognathic surgery has complications as well such as neurovascular problems, bleeding, infection, incorrect osteotomy and its consequences, traumatization of teeth, impaired bone healing, unbalanced occlusion, temporomandibular joint problems and consequently unfavorable esthetics [5].

Khadka et al. [6] evaluated the changes in quality of life following orthognathic surgery using 22-item orthognathic quality of life questionnaire (OQLQ) preoperatively and 6–8 months postoperatively. They found that orthognathic surgery had a positive effect on quality of life of patients regardless of the type of deformity. Lee et al. [7] assessed the impact of orthognathic surgery on quality of life at baseline (presurgical phase), 6 weeks postoperatively and 6 months postoperatively. Generic health-related quality of life was evaluated using the 36-item Short Form Health Survey. The generic oral health-related quality of life was evaluated by the 14-item Short Form Oral Health Impact Profile (OHIP-14), while condition-specific quality of life was evaluated using the 22-item OQLQ. They reported significant changes in quality of life following orthognathic surgery. A marked but temporary deterioration was noted in many aspects related to general wellbeing in the early postoperative period. Significant improvement was documented at 6 months. They confirmed the usefulness of comprehensive assessment of quality of life using generic health, generic oral health, and condition-specific approaches.

The objectives of orthognathic surgery include improvement of morphology and function of occlusion as well as psychological perception and quality of life of patients [8]. Since the perception of beauty widely varies among different populations and racial and ethnic groups, the effect of class III skeletal malocclusion and positive effects of orthognathic surgery on the quality of life of class III patients must be separately evaluated in different populations. Also, considering the significance of the appearance of the teeth, class of malocclusion and facial esthetics in social communications and their psychological impact, it is imperative to assess the magnitude of the effect of facial disharmony on the quality of life of patients. Moreover, it is important to find out whether the conduction of orthognathic surgery can improve the psychological status and oral health-related quality of life (OHRQoL) of patients. Considering the lack of such comprehensive studies on the Persian population and the significance of orthognathic surgery for improvement of esthetics and function, this study aimed to assess the OHRQoL of patients with class III skeletal malocclusion before and after orthognathic surgery.

## Materials and methods

This descriptive quasi-experimental study evaluated Persian adult patients with class III skeletal malocclusion presenting to a private orthodontic clinic in Kermanshah city, Iran. The data were collected by our research team comprising of an oral and maxillofacial surgeon, two orthodontists and a dental student. The study was approved by the Ethics Committee of Kermanshah University of Medical Sciences (approval number: IR.KUMS.REC.1396.435).

The following three questionnaires were used for data collection:

The OHIP-14 was first used to assess the OHRQoL of patients as our primary outcome measure. According to John et al., [9], one summary score was calculated instead of reporting separate scores for the seven domains of OHIP-14.

Higher scores indicated poorer oral-health related quality of life. The validity and reliability of the Farsi version of this questionnaire have been previously confirmed [10].

The OQLQ is a suitable tool for the assessment of orthognathic quality of life. It has 22 questions covering four domains of the orthognathic quality of life including social aspects, dentofacial esthetics, oral function, and awareness of dentofacial esthetics [11]. Selection of answer choice 1 means that the issue covered by the statement slightly bothers the patient. Choice 4 means that the issue covered in the statement bothers the patient a lot. Choices 2and 3 rank in between the two extremes. Selection of the choice “N/A” means that the issue covered in the statement does not apply to the patient or if it does, it does not bother the patient at all. The final score of the questionnaire is the sum of scores given to each question and can range from 0 to 88. Lower scores indicate better and higher scores indicate poorer quality of life [11].

The minimum sample size was calculated to be 66 patients (*n* = 22 in each group) according to a study by Mousoulea et al., [3] assuming the standard deviation of the OHIP-14 score to be 4.62 and 6.08 in the control and monomaxillary groups, respectively, alpha = 0.05, and study power of 90%. To increase the reliability of the results, a minimum of 25 patients were evaluated in each group. Patients were selected using convenience sampling. A total of 112 class III patients were evaluated in three groups. Group 1 included 25 class III patients who sought orthodontic treatment and presented for primary examination and orthosurgical treatment planning. Group 2 included 62 class III patients who had already undergone orthodontic treatment in private offices for the purpose of preparation for orthognathic surgery, and their orthognathic surgery was scheduled for the following month. Group 3 included 25 class III patients who had undergone orthognathic surgery and 2 to 6 months had passed since removal of their orthodontic appliances (they were in the retention phase). The inclusion criteria were as follows:

1. Absence of developmental syndromes
2. Absence of cleft lip or palate or history of trauma
3. Age between 18 to 30 years
4. Mental and psychological health
5. No history of orthodontic treatment
6. Class III malocclusion

Prior to the onset of orthodontic treatment, patients were interviewed, their chief complaint was recorded and a thorough clinical examination was performed. The soft tissue profile was determined and dental occlusion was evaluated. All patients signed informed consent forms prior to participation in the study. Patients were provided with a demographic questionnaire, the OHIP-14, and OQLQ, as well as instructions on how to fill out the questionnaires. Patients filled out the questionnaires under the supervision of the examiner.

Data were analyzed using descriptive and inferential statistics. Normal distribution of data was evaluated using the Kolmogorov-Smirnov test. For normally distributed data, multiple comparisons were performed using ANOVA. Independent samples t-test was used for between-group comparisons. Pairwise comparisons were carried out using the Tukey’s post hoc test. Between-group comparisons were performed using the Mann Whitney U test for the data which were not normally distributed. Multiple comparisons were performed using the Kruskal-Wallis test, and pairwise comparisons were carried out using the Dunn test with Bonferroni adjustment. All statistical analyses were carried out using SPSS version 18 (SPSS Inc., IL, USA). Level of significance was set at 0.05.

## Results

A total of 112 patients participated in this study; out of which, 39 (34.8%) were males and 73 (65.2%) were females. The mean age of participants was 23.18 ± 5.22 years.

The three groups were not significantly different in terms of gender distribution (chi-square test, *P* = 0.854), marital status (Monte Carlo chi-square, *P* = 0.242), level of education (chi-square, *P* = 0.103) or occupation (Monte Carlo chi-square, *P* = 0.4). The three groups were not significantly different in terms of the mean age either (ANOVA, *P* = 0.309).

The OHIP-14 summary score changed from 14.25 “prior to orthodontic treatment” to 23.37 “prior to surgery and during orthodontic treatment” to 5.36 “after surgery”. These OHIP-14 changes were statistically significant (*P* < 0.001).

Table 1 shows the mean and standard deviation (SD) of OQLQ domains in the three groups. Dentofacial esthetics changed from 13.80 “prior to orthodontic treatment” to 17.61 “prior to surgery and during orthodontic treatment” to 6.44 “after surgery” and these changes were statistically significant (*P* < 0.001). Oral function changed from 11.80 “prior to orthodontic treatment” to 15.32 “prior to surgery and during orthodontic treatment” to 5.96 “after surgery” and these changes were statistically significant (*P* < 0.001). Awareness of dentofacial esthetics changed from 9.80 “prior to orthodontic treatment” to 13.50 “prior to surgery and during orthodontic treatment” to 12.40 “after surgery” and these changes were statistically significant (*P* < 0.001). OQLQ score changed from 55.96 “prior to orthodontic treatment” to 70.02 “prior to surgery and during orthodontic treatment” to 45.96 “after surgery” and these changes were statistically significant (*P* < 0.001).

**Table 1:** Mean and standard deviation of OQLQ domains in the three groups

		Prior to Orthodontic Treatment	Prior to Surgery (during orthodontic treatment)	After Surgery	*P*-value^a^
Social aspects	Mean	20.56	23.58	21.16	0.096
	SD	5.22	6.25	8.47	
Dentofacial esthetics	Mean	13.80	17.61	6.44	< 0.001
	SD	3.21	5.38	2.62	
Oral function	Mean	11.80	15.32	5.96	< 0.001
	SD	2.61	4.64	1.93	
Awareness of Dentofacial esthetics	Mean	9.80	13.50	12.40	< 0.001
	SD	2.14	4.13	3.67	
OQLQ	Mean	55.96	70.02	45.96	< 0.001
	SD	8.18	16.19	10.89	

^a^ ANOVA followed by Tukey’s test

*SD* Standard deviation

Table 2 shows the mean and SD of OHIP-14 summary score separately in males and females in the three groups. The mean summary score of OHIP-14 in males was higher than that in females “before orthodontic treatment” and “after surgery”, while the mean summary score in females was greater than that in males “prior to surgery”. However, the difference in this regard was not significant between males and females in any group (*P* > 0.05).

**Table 2:** Mean and standard deviation of OHIP-14 summary score separately in males and females in the three groups

OHIP-14 Summary score	Gender	Prior to Orthodontic Treatment		Prior to Surgery (during orthodontic treatment)		After Surgery	
		Mean	SD	Mean	SD	Mean	SD
	Male	20.00	12.55	20.78	10.44	7.13	6.36
	Female	11.94	5.44	24.90	10.25	4.53	2.81
	*P*-value	0.119		0.135		0.165	

*SD* Standard deviation

Table 3 presents the mean and SD of OQLQ domains separately in males and females in the three groups. The difference in social aspects was not significant between males and females (*P* > 0.05). The mean scores of dentofacial esthetics (*P* = 0.007) and oral function (*P* = 0.018) in females were greater than those in males “prior to surgery”. The mean score of awareness of dentofacial esthetics and the mean questionnaire score (*P* = 0.007) in females were greater than those in males “prior to surgery” (*P* = 0.009).

**Table 3:** Mean and standard deviation of OQLQ parameters separately in males and females in the three groups

Domain	Gender	Prior to Orthodontic Treatment		Prior to Surgery (during orthodontic treatment)		After Surgery	
		Mean	SD	Mean	SD	Mean	SD
Social aspects	Male	22.50	6.34	22.48	6.46	19.38	8.52
	Female	19.76	4.60	24.23	6.11	22.00	8.57
	*P*-value	0.343		0.290		0.481	
Dentofacial esthetics	Male	15.13	3.52	15.26	5.02	7.75	3.77
	Female	13.18	2.96	19.00	5.16	5.82	1.67
	*P*-value	0.162		0.007		0.203	
Oral function	Male	11.38	2.33	13.52	3.60	66.38	3.11
	Female	12.00	2.78	16.38	4.89	5.76	1.09
	*P*-value	0.588		0.018		0.471	
Awareness of Dentofacial esthetics	Male	10.50	1.93	11.70	4.12	12.88	3.48
	Female	9.47	2.21	14.56	3.80	12.18	3.84
	*P*-value	0.271		0.009		0.667	
OQLQ	Male	59.25	9.87	62.69	14.11	46.38	10.04
	Female	54.41	7.06	74.18	16.05	45.76	11.56
	*P*-value	0.173		0.007		0.899	

*SD* Standard deviation

The OHIP difference (follow-up minus baseline) in this study was 9.16.

## Discussion

Jaw discrepancy can negatively affect the quality of life [12]. Facial appearance affects the self-image of individuals [13] and their life expectancy [14]. Many studies have evaluated the effects of malocclusion on the quality of life of adolescents and have shown that malocclusion is associated with higher level of dissatisfaction with facial appearance [13, 15, 16].

The OHIP-14, invented by Slade [17] is commonly used for assessment of the effect of oral health on the quality of life. We evaluated the quality of life of patients before orthodontic treatment, prior to surgery (1 month before orthognathic surgery) and after surgery at 2 to 6 months following appliance removal and in the retention phase using the OHIP-14 and OQLQ. Nicodemo et al. [18] evaluated the effect of orthognathic surgery on quality of life of class III patients and concluded that orthognathic surgery positively affected the quality of life of both males and females in physical and social aspects. However, they used the SF-36 questionnaire and evaluated patients before and after surgery (not before orthodontic treatment).

Regarding the summary score of OHIP-14, the lowest and the highest scores were acquired by patients after and before surgery, respectively. It shows that patient satisfaction with oral function is the lowest before and the highest after orthognathic surgery. Kilinc and Ertas [4] also reported a significant difference in this respect but this difference was not significant in the study by Lee et al. [7].

The OQLQ was also used in our study with four domains of social aspects, dentofacial esthetics, oral function, and awareness of dentofacial esthetics. The three groups were not significantly different in terms of social aspects. This domain evaluates the significance of the opinion of the others about the patient. Kilinc and Ertas [4] and Lee et al. [7] did not find a significant difference in this respect either. Dentofacial esthetics domain shows the shyness of patients and their satisfaction with their own appearance. The difference in this respect was significant among the three groups in our study and the lowest mean score was noted after surgery while the highest mean score was recorded prior to surgery. This finding shows the positive effect of surgery on this domain. Lee et al. [7] also reported a reduction in this domain after surgery. The difference in oral function was significant among the three groups such that the lowest mean was noted after and the highest mean was recorded before surgery. This finding highlights the positive effect of surgery on the masticatory function. Kilinc and Ertas [4] and Lee et al. [7] did not find a significant difference in this respect. A significant difference was also noted in awareness of dentofacial esthetics among the three groups and the lowest mean was noted in patients prior to orthodontic treatment. Kilinc and Ertas [4] and Lee et al. [7] did not find a significant difference in this respect. Regarding the total score of OQLQ, the lowest total score was acquired by patients after surgery while the highest score was acquired by patients prior to surgery. Kilinc and Ertas [4] and Lee et al. [7] did not find a significant difference in this regard.

The current findings indicate that in general, the OHRQoL of patients with class III malocclusion increases after orthognathic surgery. Posnick and Wallace [19] concluded that orthognathic surgery is associated with high level of patient satisfaction. Pahkala and Kellokoski [20] reported that orthognathic surgery decreased the symptoms of temporomandibular disorders and pain and improved facial esthetics and chewing. Moreover, most patients were satisfied with the treatment outcome. Nicodemo et al. [18] concluded that orthognathic surgery has a positive effect on the quality of life of both males and females and improves physical and social aspects. Esperao et al. [21] discussed that orthognathic surgery positively affects the quality of life. Imani et al. [22] evaluated the effect of orthodontic intervention on mental health and body image and concluded that orthodontic treatment significantly improves the mental health status and multidimensional attitudes towards body image.

In general, level of satisfaction of patients decreases prior to surgery, which may be due to elimination of dental compensations or lack of knowledge of patients about the phases of orthodontic treatment. Raising awareness in this respect may increase the level of satisfaction of these patients. This issue is in need of further investigation in future studies.

Our results showed no significant difference between males and females in OHIP-14 summary score in any of the three groups.

Regarding the difference between males and females in the OQLQ domains, the mean scores of dentofacial esthetics, oral function, awareness of dentofacial esthetics and overall score of the questionnaire before surgery in females were higher than those in males. It means that the orthognathic quality of life in males was higher than that in females prior to surgery.

Locker et al., [23] and Reissmann et al. [24] interpreted the change in OHIP scores in relationship to the minimal important difference (MID). In this study, we mainly focused on statistical significance of the findings and did not calculate the MID, which was a limitation of our study. However, the OHIP difference (follow-up minus baseline) in our study was found to be 9.16. The MID reported by Reissmann et al. [24] was 2. Since the OHIP difference in our study was larger than the MID reported by Reissmann et al., [24] it may be concluded that the intervention had a clinically meaningful effect on OHRQoL of patients.

Absence of a control group was another limitation of this study. Future studies with a larger sample size, longer follow-up and a control group are recommended to further elucidate this topic. Also, further studies are recommended to assess patients during the entire course of treatment.

## Conclusion

Within the limitations of this study, the results showed that orthognathic surgery in skeletal class III patients improves their quality of life, satisfaction with different aspects of quality of life, self-confidence, and oral function. Also, the results showed that removal of dental compensations decreased the satisfaction rate of patients while orthognathic surgery improved the quality of life and satisfaction of patients. Females were more sensitive to their esthetic appearance and oral function than males, and class III skeletal malocclusion was more accepted by males. The quality of life and satisfaction of patients significantly improved after surgery and this improvement was significantly greater in females than males in many aspects.

## Data Availability

The datasets used and/or analysed during the current study available from the corresponding author on reasonable request.

## Abbreviations

OHIP-14: Oral health impact profile-14
OHRQoL: Oral health-related quality of life
OQLQ: Orthognathic quality of life questionnaire
SD: Standard deviation
